# CD4+ T-Cell Help Is Required for Effective CD8+ T Cell-Mediated Resolution of Acute Viral Hepatitis in Mice

**DOI:** 10.1371/journal.pone.0086348

**Published:** 2014-01-21

**Authors:** Tanja Trautmann, Jan-Hendrik Kozik, Antonella Carambia, Kirsten Richter, Timo Lischke, Dorothee Schwinge, Hans-Willi Mittrücker, Ansgar W. Lohse, Annette Oxenius, Christiane Wiegard, Johannes Herkel

**Affiliations:** 1 Department of Medicine I, University Medical Center Hamburg-Eppendorf, Hamburg, Germany; 2 Institute of Microbiology, Swiss Federal Institute of Technology Zurich, Zürich, Switzerland; 3 Institute of Immunology, University Medical Center Hamburg-Eppendorf, Hamburg, Germany; Institut Pasteur, France

## Abstract

Cytotoxic CD8+ T cells are essential for the control of viral liver infections, such as those caused by HBV or HCV. It is not entirely clear whether CD4+ T-cell help is necessary for establishing anti-viral CD8+ T cell responses that successfully control liver infection. To address the role of CD4+ T cells in acute viral hepatitis, we infected mice with Lymphocytic Choriomeningitis Virus (LCMV) of the strain WE; LCMV-WE causes acute hepatitis in mice and is cleared from the liver by CD8+ T cells within about two weeks. The role of CD4+ T-cell help was studied in CD4+ T cell-lymphopenic mice, which were either induced by genetic deficiency of the major histocompatibility (MHC) class II transactivator (CIITA) in CIITA−/− mice, or by antibody-mediated CD4+ cell depletion. We found that CD4+ T cell-lymphopenic mice developed protracted viral liver infection, which seemed to be a consequence of reduced virus-specific CD8+ T-cell numbers in the liver. Moreover, the anti-viral effector functions of the liver-infiltrating CD8+ T cells in response to stimulation with LCMV peptide, notably the IFN-γ production and degranulation capacity were impaired in CIITA−/− mice. The impaired CD8+ T-cell function in CIITA−/− mice was not associated with increased expression of the exhaustion marker PD-1. Our findings indicate that CD4+ T-cell help is required to establish an effective antiviral CD8+ T-cell response in the liver during acute viral infection. Insufficient virus control and protracted viral hepatitis may be consequences of impaired initial CD4+ T-cell help.

## Introduction

Viral infections of the liver are a major cause of illness and death worldwide. In particular, virus-induced hepatitis, leading to chronic disease in hundreds of millions of people, is one of the most common causes of liver cirrhosis and liver cancer [1]. After infection with hepatitis viruses, some individuals are able to clear the infection, whereas others remain infected and manifest chronic liver inflammation [2]–[5]. The ability to clear viral liver infection is determined both by viral and host factors, but the adaptive antiviral immune response is believed to be the most important determinant [2]–[6]. Indeed, patients that spontaneously clear the infection during acute hepatitis, show a vigorous and polyclonal T-cell response, whereas chronically infected patients seem to have delayed, transient or pauciclonal T-cell responses [6].

It is widely accepted that CD8+ T cells are the major effector cells that mediate viral clearance from the liver by removal of infected cells; the role of CD4+ T cells in viral hepatitis is less clear [6]–[8]. On the one hand, relapse of HCV infection after initial control was associated with a loss of the antiviral CD4+ T-cell response [9]. Moreover, re-challenge of chimpanzees that had cleared a previous viral infection was poorly controlled in the absence of a functional CD4+ T-cell response [10], [11]. Furthermore, several studies (reviewed in [6]) indicate an association between a broad initial antiviral CD4+ T-cell response and viral clearance. However, on the other hand, depletion of CD4+ T cells in an early phase of HBV infection did not influence duration and outcome of acute HBV infection in a chimpanzee study [12]. Moreover, recent findings indicate that the early presence of a broad anti-HCV CD4+ T-cell response does not determine whether HCV is cleared or persists [13]. Furthermore, at least in certain virus infections, type I IFN is able to promote anti-viral CD8+ T-cell responses without dependence on CD4+ T cell help [14]. Thus, the role of CD4+ T cells in the early phase of viral liver infection remains to be clarified.

To address this issue in a controlled study, we used a mouse model of viral hepatitis induced by infection with Lymphocytic Choriomeningitis Virus (LCMV) of the strain WE. Infection with a high inoculum (10^6^ FFU) of LCMV-WE causes acute hepatitis [15], [16]; the virus is usually cleared by wild-type mice within about two weeks. LCMV hepatitis is a useful model for human hepatitis virus infections, in so far as LCMV-WE, similar to human hepatitis viruses, causes a non-cytopathic infection, in which the liver damage is mediated almost entirely by the antiviral immune response [16]. Also in LCMV infection, CD8+ T cells are essential for the elimination of the virus [17], [18]. It is believed that CD4+ T cells are required for sustaining CD8+ T-cell responses, thus preventing CD8+ T-cell exhaustion and chronic LCMV infection [19], [20]. Indeed, administration of CD4+ T cells can resurrect an already exhausted CD8+ T-cell response [21]. However, CD4+ T cells do not seem to be required for the initiation of the CD8+ T-cell response to LCMV and the control of acute LCMV infection [22]–[24].

To study the role of CD4+ T cells in LCMV-induced hepatitis, we compared the outcome of LCMV infection in wild-type C57BL/6 mice that have normal CD4+ T-cell numbers with that in CD4+ T cell-lymphopenic C57BL/6 mice. CD4+ T cell-lymphopenia was either induced by anti-CD4 antibody-mediated cell depletion or by genetic deficiency of the major histocompatibility (MHC) class II transactivator (CIITA) in CIITA−/− mice [25]. CIITA is the master regulator of MHC class II expression in peripheral tissues [25]. In CIITA−/− mice, CD4+ T cells develop, but do not expand, as they are not appropriately stimulated in the periphery [25]. We demonstrate that CD4+ T cell-lymphopenic mice manifested prolonged LCMV infection of the liver, as compared to CD4+ T cell-replete mice. Moreover, CD4+ T cell-lymphopenic mice manifested significantly reduced numbers of liver-infiltrating cytotoxic CD8+ T cells. In particular, the numbers of functional LCMV-specific CD8+ effector T cells were significantly reduced in the livers of CD4+ T cell-lymphopenic mice. The reduction of antiviral CD8+ T-cell effector functions was not associated with elevated exhaustion markers in the liver. These findings indicate that CD4+ T-cell help is required to establish efficient virus control by CD8+ T cells in the liver during acute LCMV infection.

## Materials and Methods

### Mice and Virus

C57BL/6 mice and CIITA−/− mice on C57BL/6 background were bred and kept at specific pathogen-free conditions in the animal facilities of the University Medical Centre Hamburg-Eppendorf. Male mice were used at the age of 8 to 15 weeks; LCMV infection was performed at the animal facilities of the Heinrich-Pette Institute, Leibniz Institute for Experimental Virology, Hamburg. All animal work has been conducted according to relevant national and international guidelines; animal experiments were approved by the review board of the State of Hamburg, Germany (Permit number 96/06 and 102/10). LCMV-WE was originally provided by Prof. R. M. Zinkernagel (University Hospital, Zürich, Switzerland) and propagated on L929 mouse fibroblasts (DSMZ-No. ACC-2). Mice were infected intravenously with 10^6^ FFU of virus, as determined by a modified protocol of the previously described Focus-Forming Assay [26]. Here, staining of Foci was conducted with the DAKO Envision Kit.

### RNA Isolation and RT-PCR

Mouse livers were snap frozen and total RNA was isolated using a NucleoSpin TriPrep Kit (Macherey-Nagel). 1 µg of RNA was used for reverse transcription using the AMV First-Strand Synthesis Kit (Roche). Quantitative real time-PCR for the LCMV Z-Protein was performed with cDNA of 0.05 µg transcribed RNA and LightCycler FastStart DNA Master SYBR Green I (Roche). Peptidylprolyl Isomerase A (PPIA) Primer Assay (Qiagen) was used for normalization according to the ΔCt method [27]. All PCRs were performed in duplicate with the primer sequences LCMV_Z_fwd (5′-CAGACACCACCTATCTTGG-3′) and LCMV_Z_rev (3′-ACCTTCAGTTTGGTTGGC-5′). Alternatively, TaqMan probes for chemokines CXCL9 (Mm 00434946_m1), CXCL10 (Mm 00445235_m1), or CXCL11 (Mm 00444662_m1) were used in TaqMan PCR (Life Technologies).

### Depletion of CD4+ T Cells

CD4+ T cell-depleting antibody (GK1.5) and rat IgG2b isotype-matched control antibody (LTF-2) were obtained from BioXCell. At days –3, –2 and –1 before LCMV infection, 0.3 mg of antibody were injected intraperitoneally into C57BL/6 wild-type mice; followed by antibody injections twice weekly after virus inoculation.

### Cell Isolation

Antiviral immune responses were analyzed in spleen and liver of infected mice. Following mechanical dissection of the spleen, splenocytes were subjected to ACK lysis. After perfusion with PBS, livers were mechanically dissected and mononuclear liver cells were obtained through isolation on a density gradient. To that end, liver cells were taken up into a layer of 5 ml 30% Percoll (GE Healthcare), covered onto a layer of 3 ml 70% Percoll, and subjected to centrifugation at 524 g for 20 min at 20°C.

### Flow Cytometry

Dead cell staining was performed with Pacific Orange Succinimidyl Ester, Triethylammonium Salt (Life Technologies) in PBS. Immunofluorescent surface staining of T cells was performed in 2% BSA with fluorochrome-conjugated antibodies to CD4, CD8, CD44, CD107a, PD-1 or IFN-γ (all from BioLegend), or with Dextramers specific for the immunodominant H-2D^b^ restricted gp33-41 (KAVYNFATC) LCMV peptide (Immudex). Stained cells were fixed overnight in 1% PFA in PBS. For staining of CD107a or intracellular IFN-γ, cells were stimulated for 4 hours with the immundominant gp33-41 (KAVYNFATC) LCMV peptide (3 µg/ml) in the presence of 1 µl/ml Golgi-Plug (IFN-γ) or 0.65 µl/ml Golgi-Stop (CD107a) (both from BD Bioscience) in Panserin 401 medium (PAN Biotec), supplemented with 1% penicillin/streptomycin and 0.5×10^4^ M β-Mercaptoethanol. For intracellular IFN-γ staining, cells were then perforated in buffer containing 2% BSA/0.5% Saponin and stained with antibody against IFN-γ. Flow cytometry was performed with an LSR II cytometer and data were analyzed with FACSDiva 6.0 Software (both BD Bioscience).

### Immunohistochemistry

Frozen liver sections were blocked with 1% BSA, 5% normal rat serum, 1∶50 Fc-Block (eBioscience) and 1∶50 mouse IgG in PBS. Staining was performed in 1% BSA and 5% normal rat serum with CD8-PE (Abcam) and VL-4 antibody from a hybridoma cell line labeled with Alexa-488 labeling kit (Life Technologies). Cell nuclei were stained with Hoechst 33258 (Life Technologies). Microscopy was performed with a Keyence BZ-9000 microscope.

### Statistics

Statistical significance of differences between two data sets was tested by the Mann-Whitney test; for comparison of multiple groups, the Kruskal-Wallis test and Dunn's post test were performed. *P* values <0.05 (*), <0.01 (**), <0.001 (***) were considered significant. Bars represent the medians.

## Results

### Defective Clearance of LCMV from Livers of CIITA−/− Mice

We first analyzed CIITA−/− mice for CD4+ and CD8+ T-cell numbers in spleen and liver in comparison to wild-type C57BL/6 mice (Figure S1). As expected, the numbers of CD4+ T cells were greatly reduced both in spleen and liver of CIITA−/− mice; nonetheless, both mouse strains showed comparably high numbers of CD8+ T cells.

**Figure 1:**
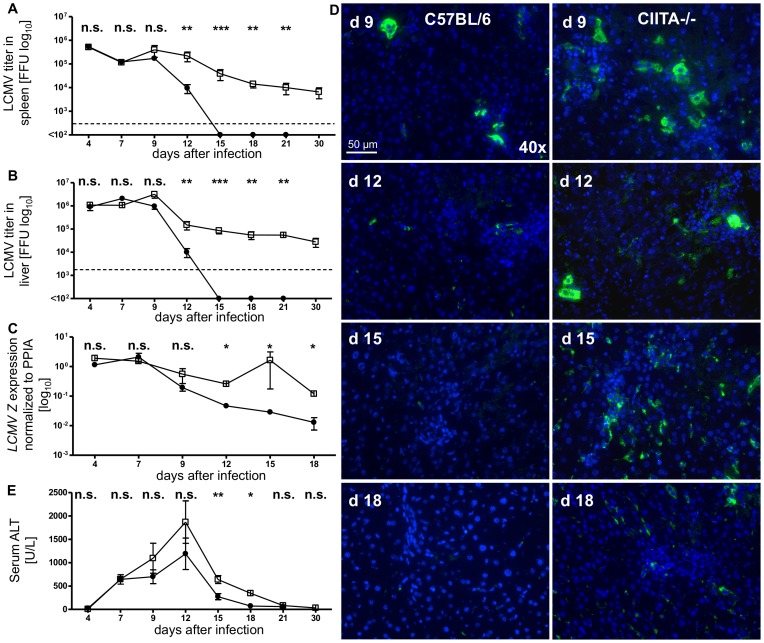
Increased and persistent LCMV infection in CIITA−/− mice. C57BL/6 and CIITA−/− mice were infected with 10^6^ Focus Forming Units (FFU) LCMV-WE. (A) At the indicated time-points after infection, spleens were homogenized and the virus titer was determined by the Focus Forming Assay. Black dots represent the mean titer of C57BL/6 mice, white squares represent the mean titer of CIITA−/− mice. The broken line represents the limit of detection. (B) At the indicated time-points after infection, the infection rate of the liver was determined as in (A). (C) At the indicated time-points after infection, the infection rate of the liver was determined by quantitative RT-PCR analysis for the LCMV Z RNA. (D) Frozen liver sections taken at the indicated time-points after infection were stained for the LCMV nucleoprotein with VL4 antibody (green); nuclei were stained with Hoechst 33258 (blue). (E) At the indicated time-points after infection, serum ALT levels were determined. The graphed lines represent the mean and s.e.m.

We then infected wild-type C57BL/6 mice and CIITA−/− mice with LCMV-WE at a dose of 10^6^ FFU. At various time-points after infection (days 4, 7, 9, 12, 15, 18, 21 and 30), we determined the virus titers in spleen (Figure 1A) and liver (Figure 1B) via Focus-Forming Assay, as well as by quantitative RT-PCR of liver RNA for the LCMV Z protein (Figure 1C). The early infection kinetics were similar in both mouse strains; however, at all time points from day 12 onwards, CIITA−/− mice manifested a significantly higher degree of liver infection than wild-type mice. Whereas wild-type mice had cleared the infection by day 15, the infection persisted in CIITA−/− mice for at least 30 days. We further confirmed these findings by histological staining of liver sections for the LCMV nucleoprotein with VL4 antibody (Figure 1D). At all time points analyzed, there were more VL4 positive hepatocytes in CIITA−/− mice than in C57BL/6 mice. Consistent with the higher and protracted viral load, CIITA−/− mice manifested increased and prolonged elevation of serum ALT levels, as compared to C57BL/6 mice (Figure 1E). Thus, numerical impairment of CD4+ T cells in acute LCMV infection seemed to prevent the clearance of LCMV-WE infection and to induce protracted viral hepatitis.

### Defective Clearance of LCMV from Livers of CD4-depleted Mice

**Figure 2:**
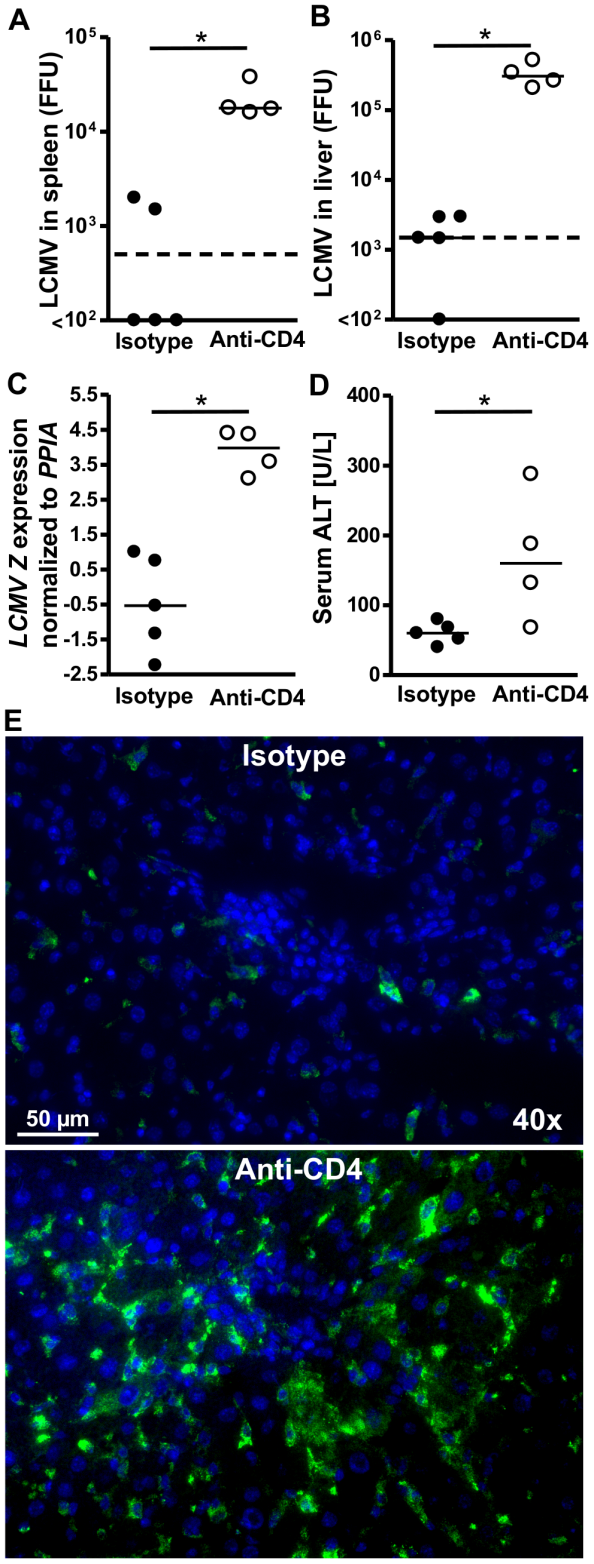
Protracted LCMV infection in livers of wild-type C57BL/6 mice depleted of CD4+ T cells. C57BL/6 mice were treated twice per week with depleting CD4 antibody (GK1.5) or isotype-matched control antibody to obtain CD4-depleted or CD4-replete mice that were subsequently infected with 10^6^ FFU LCMV-WE. All samples were taken at day 18 after infection. (A) Spleens were homogenized and the virus titer was determined by FFA. Each dot (black: C57BL/6 mice; white: CIITA−/− mice) represents the average FFU of one sample tested in duplicate; the broken line represents the limit of detection. (B) Livers were homogenized and the infection rate was determined as in (A). (C) The infection rate of the livers was determined by quantitative RT-PCR analysis for the LCMV Z RNA. (D) Serum ALT levels were determined. (E) Frozen liver sections were stained for the LCMV nucleoprotein with VL4 antibody (green); nuclei were stained with Hoechst 33258 (blue).

To confirm that the observed impairment of virus control in CIITA−/− mice was indeed caused by CD4+ T cell-lymphopenia and not by some other effect mediated by CIITA deficiency, we infected wild-type C57BL/6 mice that had been depleted of CD4+ T cells with anti-CD4 antibody (GK1.5). As control, C57BL/6 mice were treated with an isotype-matched control antibody. The efficiency of CD4+ T-cell depletion was confirmed by flow cytometry (Figure S2 A and B). At day 18 after LCMV infection, we determined the virus titers in spleen (Figure 2A) and liver (Figure 2B) by Focus-Forming Assay, as well as by quantitative RT-PCR for the LCMV Z protein (Figure 2C). Consistent with the results obtained in CIITA−/− mice, we found that CD4+ T cell-depleted mice, at day 18 after infection, still manifested significantly higher viral titers in the liver, whereas control IgG-treated animals had cleared the virus. Moreover, CD4+ cell depleted mice showed overt hepatitis, as indicated by significantly elevated serum ALT levels (Figure 2D). The protracted infection of CD4+ T cell-depleted mice was confirmed by histological staining of liver sections for the LCMV nucleoprotein with the VL4 antibody (Figure 2E), clearly showing more VL4-stained hepatocytes in CD4+ T cell-depleted mice. These findings confirmed that the presence of CD4+ T cells during acute LCMV hepatitis is required for effective virus control.

### Impaired LCMV-specific CD8+ T cell Response in Infected Livers of CIITA−/− Mice

**Figure 3:**
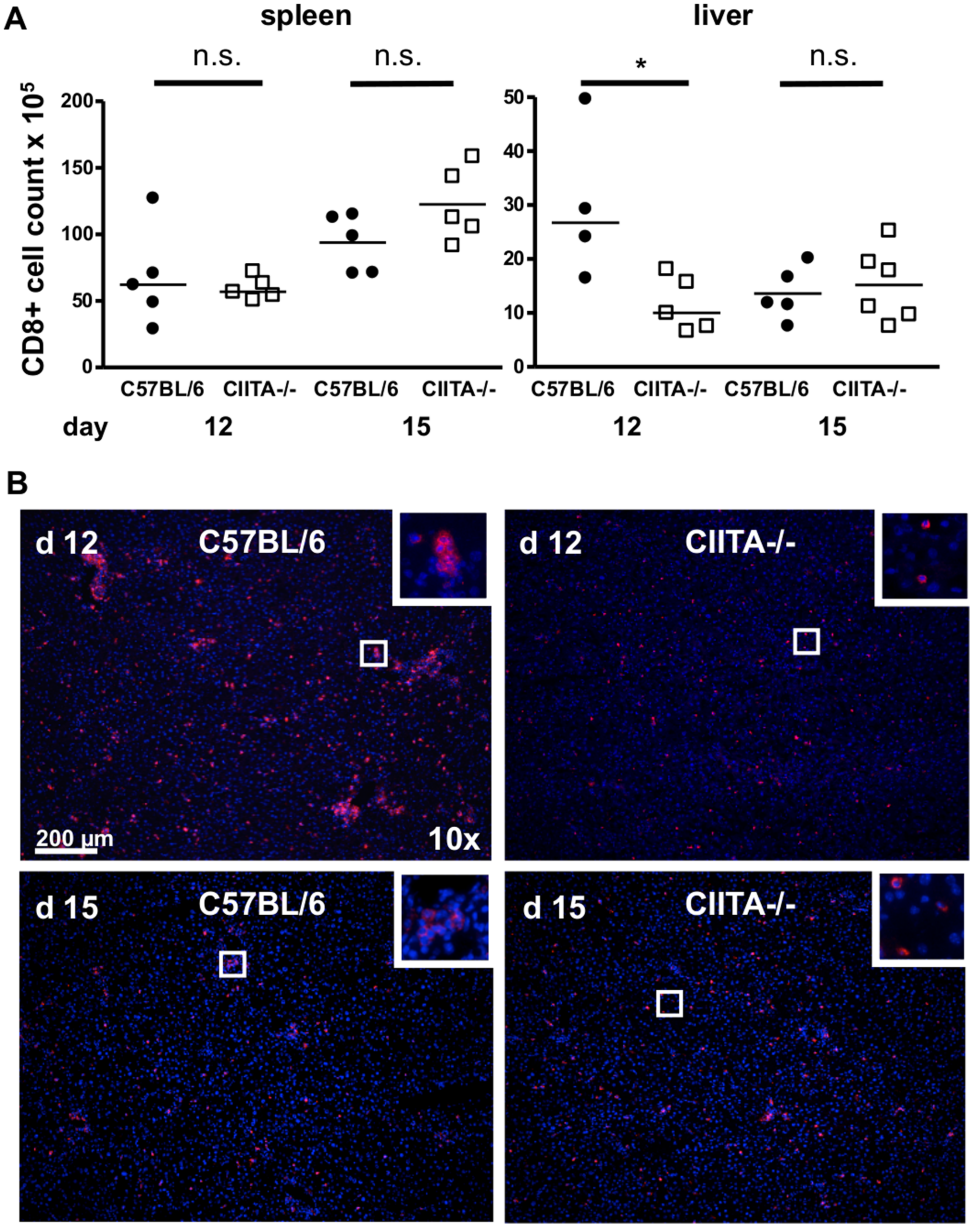
Reduced numbers of CD8+ T cells in the livers of LCMV-infected CIITA−/− mice. C57BL/6 and CIITA−/− mice were infected with 10^6 ^FFU LCMV-WE. (A) At day 12 or 15 after infection, the numbers of CD8+ T cells in spleen and liver of C57BL/6 and CIITA−/− mice were determined. Each dot represents the absolute number of CD8+ T cells per spleen or liver of one individual mouse. (B) Frozen liver sections taken at day 12 or 15 after infection were stained for CD8 T cells (red); nuclei were stained with Hoechst 33258 (blue).

Since viral clearance is predominantly mediated by CD8+ T cells, we next analyzed whether the inability of CD4+ T cell-lymphopenic mice to control LCMV infection was associated with altered frequencies of CD8+ T cells in the liver. We determined the overall numbers of CD8+ T cells in infected spleen and liver of wild-type and CIITA−/− mice in the course of LCMV infection. At days 7 and 9 after LCMV infection, the CD8+ T cell numbers in the liver were equally low both in C57BL/6 mice and CIITA−/− mice (Figure S3). At day 12, however, CD8+ T cell numbers in the livers of C57BL/6 greatly increased, whereas the numbers of CD8+ T cells in the livers of CIITA−/− mice were significantly lower (Figure 3A). At day 15, the CD8+ T-cell numbers were similarly low in both mouse strains; however, at this time point, C57BL/6 wild-type mice had already cleared the infection (Figure 3A). Of note, the CD8+ T-cell paucity seemed to be specific to the liver, since in the spleen, CD8+ T-cell numbers increased from day 12 to day 15 in both mouse strains. We confirmed this finding by histological staining of CD8+ T cells in liver sections of infected mice, showing greatly decreased numbers of CD8+ T cells in CIITA−/− livers at day 12 and equally low CD8+ T-cell numbers in wild-type and CIITA−/− livers at day 15 after infection (Figure 3B). These findings indicated that CD8+ T cells were either ineffectively recruited to infected livers of CIITA−/− mice or that the liver-infiltrating CD8+ T cells were not expanded in the absence of CD4+ T cells.

**Figure 4:**
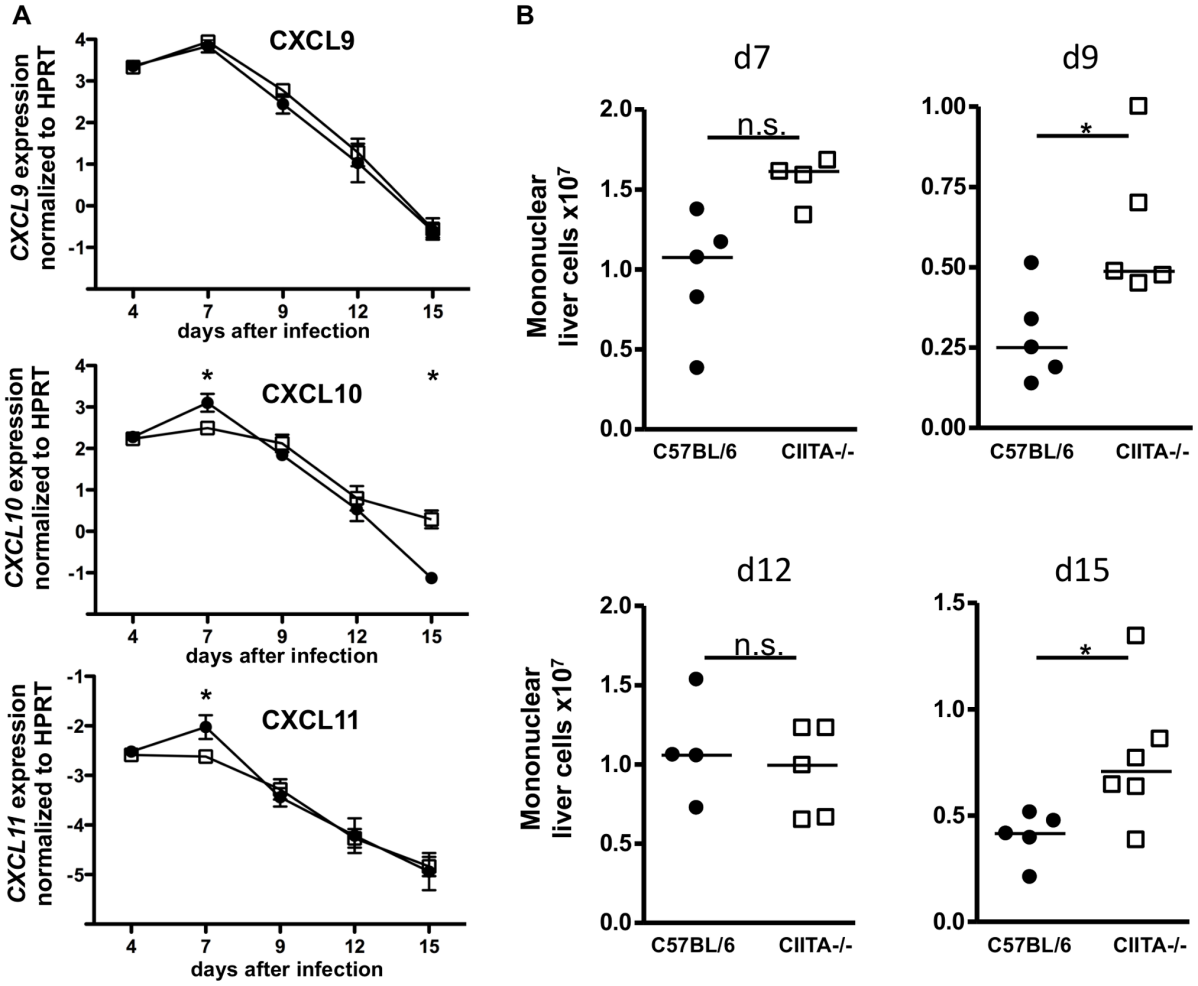
Preserved chemokine production and non-specific leukocyte recruitment to livers of infected CIITA−/− mice. C57BL/6 and CIITA−/− mice were infected with 10^6^ FFU LCMV-WE. (A) At the indicated time-points after infection, expression of the CXCR3 chemokine ligands CXCL9, CXCL10 and CXCL11 in infected livers were determined by qPCR. Black dots represent C57BL/6 mice, white squares represent CIITA−/− mice. (B) At the indicated time-points after infection, the numbers of leukocytes in infected livers were determined. Each dot represents the absolute number of liver-infiltrating mononuclear cells of one individual mouse.

As CD8+ T cell recruitment to inflamed liver mainly depends on the CXCR3 chemokine ligands CXCL9, CXCL10 and CXCL11 [28], [29], it was possible that the paucity of CD8+ T cells in LCMV-infected livers of CIITA−/− mice were due to reduced chemokine production. We therefore analyzed expression of these chemokines in livers of infected mice at various time-points (Figure 4A), but did not find major differences between the expression levels of CXCL9, CXCL10 or CXCL11 in C57BL/6 mice and CIITA−/− mice. Thus, CD8+ T cell paucity in infected livers of CIITA−/− mice was unlikely to be explained by recruitment defects. Indeed, at various time-points after LCMV-infection, the total numbers of leukocytes that had been recruited to the livers of CIITA−/− mice were at least as high as those that had been recruited to C57BL/6 livers (Figure 4B).

**Figure 5:**
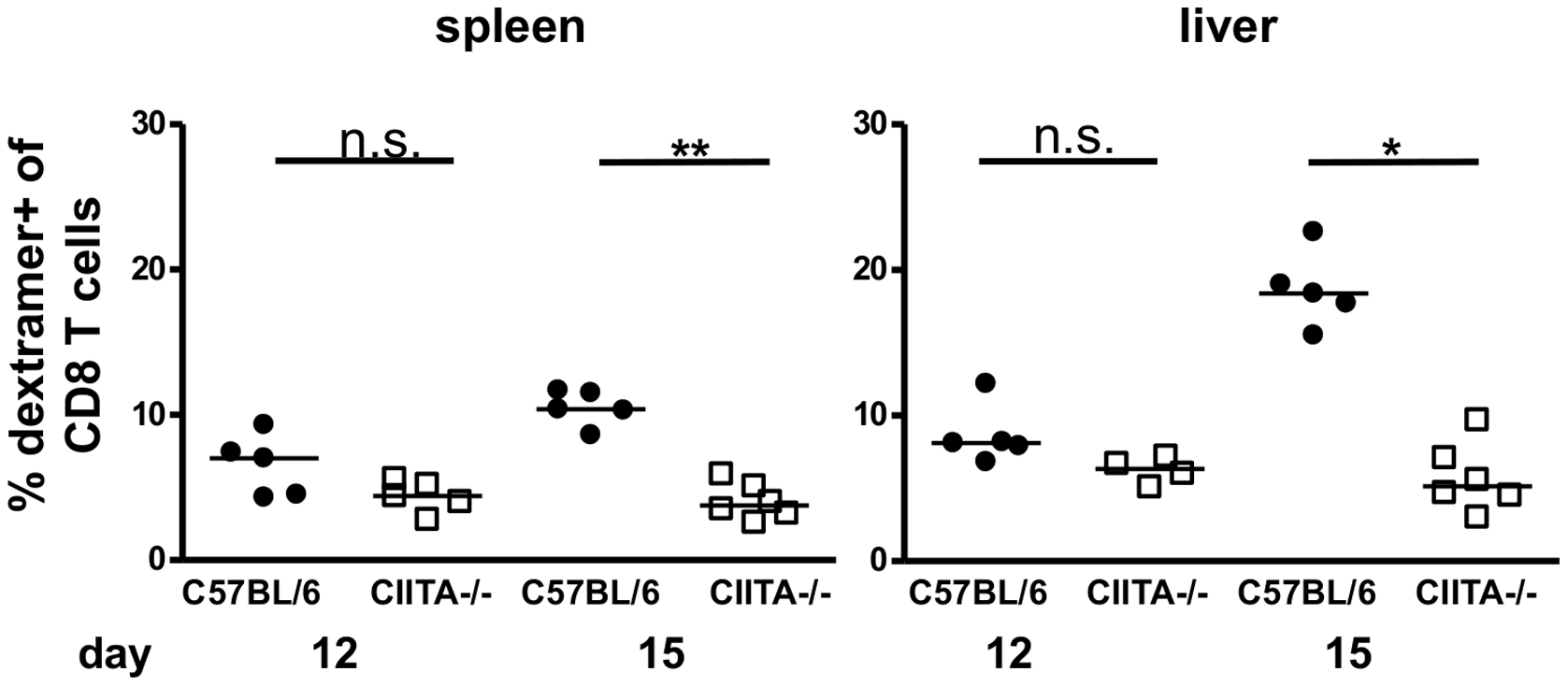
Reduced numbers of LCMV-specific CD8+ T cells in the livers of LCMV-infected CIITA−/− mice. C57BL/6 and CIITA−/− mice were infected with 10^6^ FFU LCMV-WE. At day 12 or 15 after infection, the numbers of LCMV-gp33 specific CD8+ T cells in spleen and liver of C57BL/6 and CIITA−/− mice were determined by staining with LCMV-gp33 loaded H-2D^b^ dextramer. Each dot represents the percentage of dextramer+ CD8+ T cells among all CD8+ T cells in spleen or liver of individual mice.

**Figure 6:**
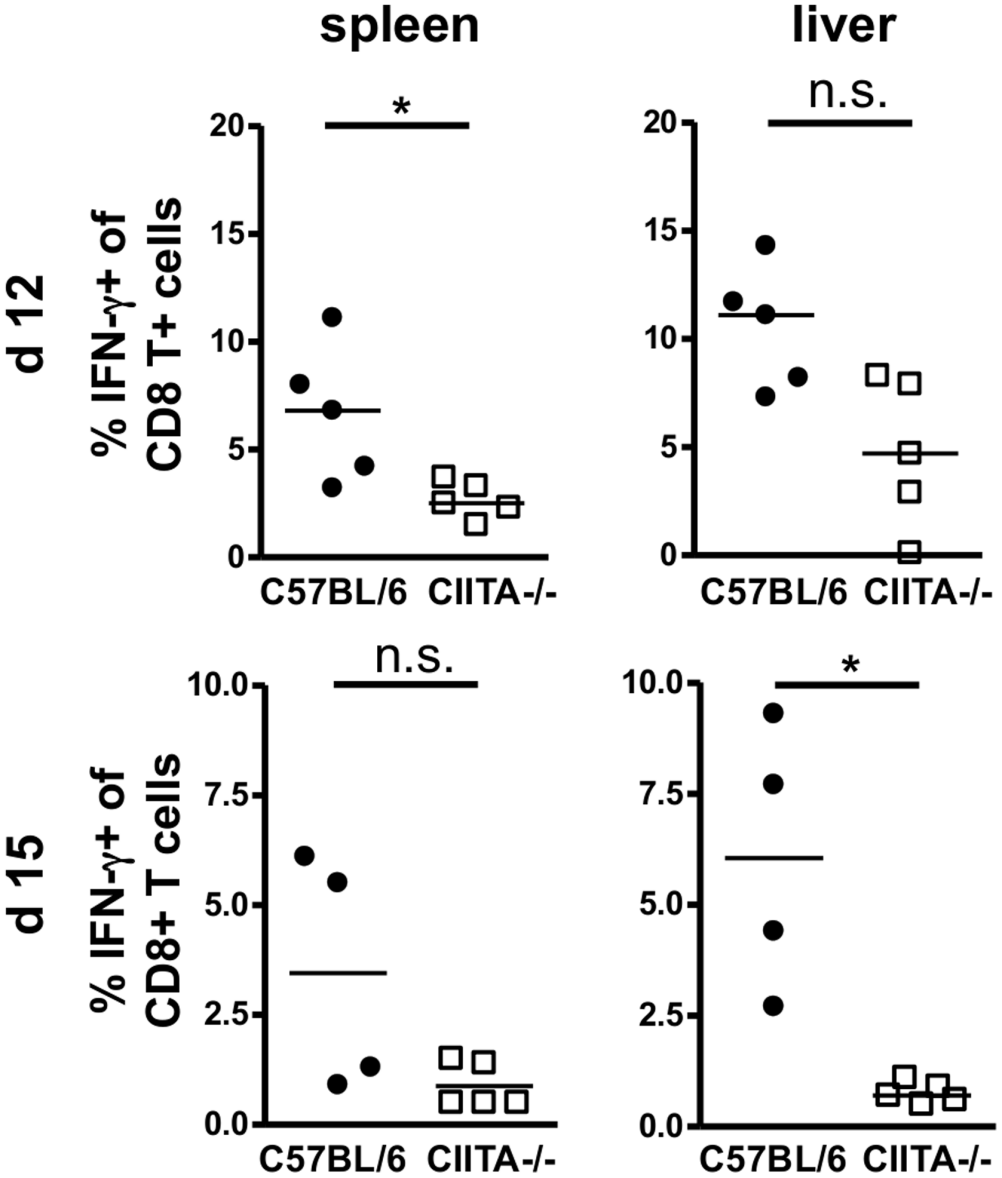
Reduced IFN-γ production by CD8+ T cells of CIITA−/− mice. C57BL/6 and CIITA−/− mice were infected with 10^6^ FFU LCMV-WE. At day 12 or 15 after infection, the effector function of LCMV-specific CD8+ T cells in spleen and liver of C57BL/6 and CIITA−/− mice was determined by intracellular staining of CD8+ T cells for IFN-γ after stimulation with LCMV-gp33 peptide. Each dot represents the percentage of IFN-γ+ CD8+ T cells among all CD8+ T cells of spleen or liver of individual mice.

**Figure 7:**
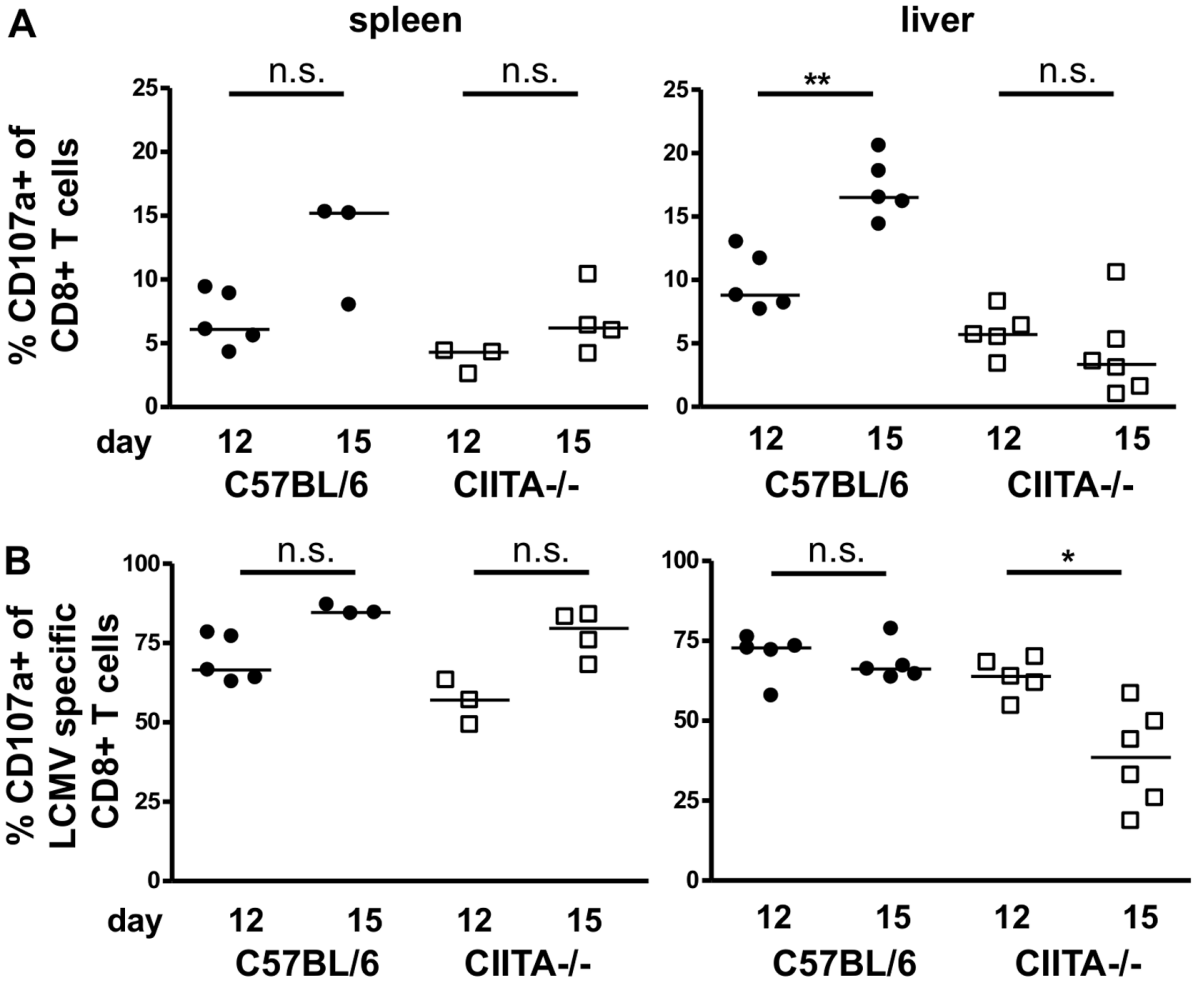
Reduced degranulation capacity of CD8+ T cells of CIITA−/− mice. C57BL/6 and CIITA−/− mice were infected with 10^6^ FFU LCMV-WE. At day 12 or 15 after infection, the degranulation capacity of CD8+ T cells (A) or LCMV-specific dextramer+ CD8+ T cells (B) in spleen and liver in response to stimulation with LCMV-gp33 peptide was determined by staining for CD107a. Each dot represents the percentage of CD107a+ CD8+ T cells among all CD8+ T cells (A) or CD107a+ dextramer+ CD8+ T cells among all CD8+ T cells (B) of spleen or liver of individual mice.

To analyze the LCMV-specific CD8+ T-cell response, we used LCMV-gp33 loaded H-2D^b^ dextramers to detect virus-specific CD8+ T cells by flow cytometry; a representative dextramer staining is shown in Figure S4. At days 7 and 9, the numbers of dextramer+ LCMV-specific CD8+ T cells were similar in the livers of infected C57BL/6 mice and CIITA−/− mice (Figure S5). However at day 15, we found significantly decreased numbers of dextramer+ LCMV-specific CD8+ T cells in the spleens and livers of CIITA−/− mice (Figure 5), indicating that CD4+ T cells seem to be required for efficient expansion or maintenance of virus-specific CD8+ T cells during acute infection. We then analyzed the antiviral effector functions of liver-infiltrating CD8+ T cells in response to stimulation with the immunodominant LCMV-gp33 peptide. To that end, we first stained liver-infiltrating CD8+ T cells for intracellular IFN-γ in response to stimulation with the LCMV-gp33 peptide (a representative staining is shown in Figure S6). At days 7 and 9, there was no difference in IFN-γ response between C57BL/6 mice and CIITA−/− mice (Figure S7). At days 12 and 15, however, we found that CIITA−/− mice manifested significantly reduced hepatic frequencies of IFN-γ producing CD8+ T cells in response to LCMV-gp33 peptide stimulation (Figure 6), which is in accordance with the finding that CIITA−/− mice had significantly lower numbers of LCMV-specific CD8+ T cells. We then assessed the ability of the CD8+ T cells to degranulate in response to stimulation with the LCMV-gp33 peptide by staining for CD107a; a representative CD107a staining is shown in Figure S8. At days 7 and 9, there was no difference in degranulation capacity between C57BL/6 mice and CIITA−/− mice (Figure S9). However, from day 12 to day 15, CD8+ T cells from C57BL/6 mice showed a significantly increased degranulation capacity in response to the stimulation with LCMV-gp33 after infection (Figure 7A), which seems to correspond with the increase from day 12 to 15 of the intrahepatic frequency of LCMV-specific T cells (see Figure 5). In contrast, CIITA−/− mice exhibited a reduced number of intrahepatic cells that were able to degranulate, both on days 12 and 15. We also assessed the degranulation capacity of LCMV-specific dextramer+ CD8+ T cells in response to the stimulation with LCMV-gp33 (Figure 7B and Figure S8), and found that the percentage of degranulated cells among the LCMV-specific CD8 T cells in the liver of C57BL/6 mice was similarly high on days 12 and 15. In contrast, the percentage of CD107a+ cells among the intrahepatic LCMV-specific dextramer+ CD8+ T cells decreased significantly in CIITA−/− mice from day 12 to day 15. These findings indicate that CD4+ T cells seem to support also the per-cell function of LCMV-specific CD8+ T cells in the liver either directly or indirectly, and thus contribute to a faster control of LCMV infection.

**Figure 8:**
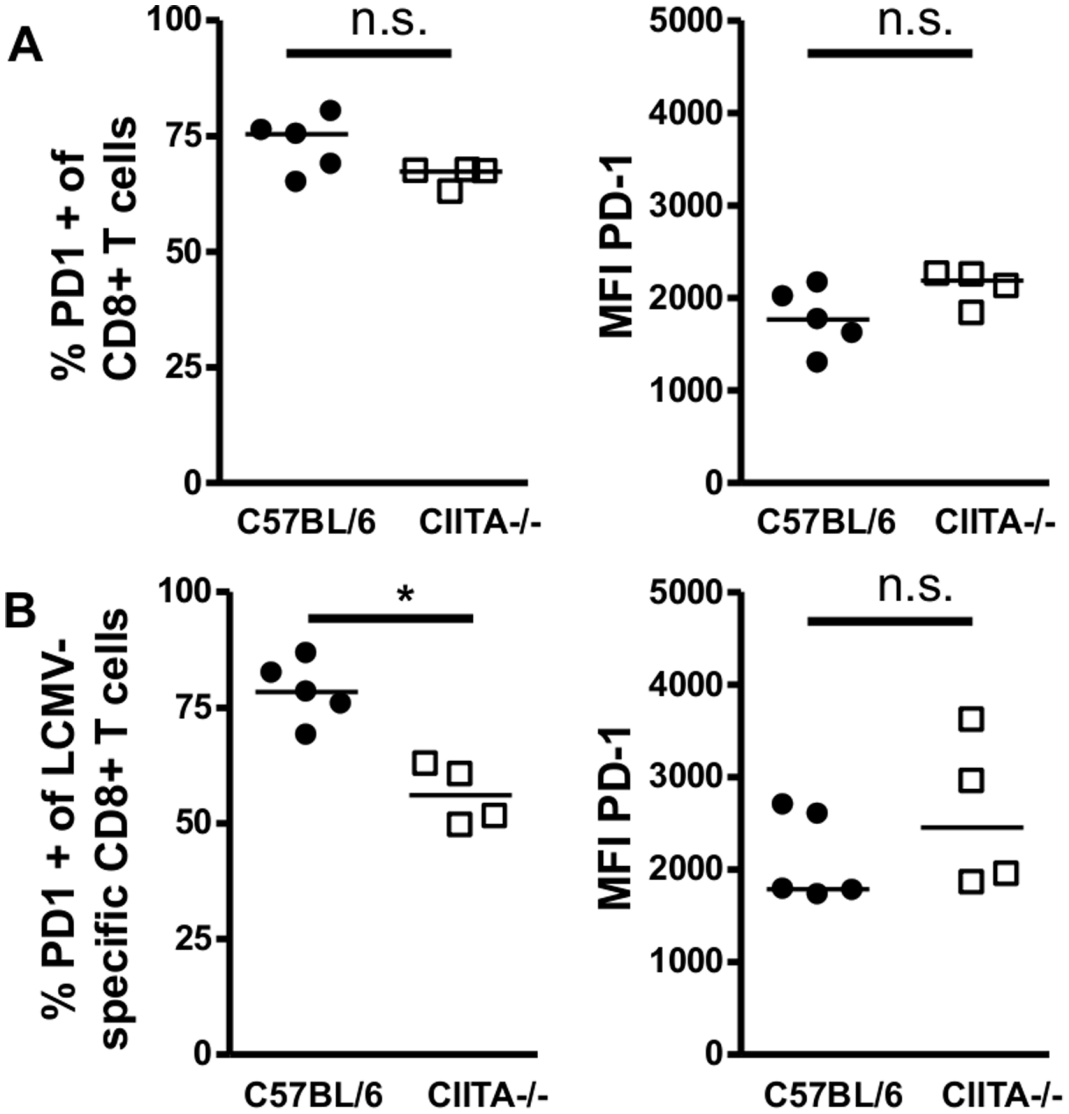
PD-1 expression of LCMV-specific CD8+ T cells in wild-type and CIITA−/− mice. C57BL/6 and CIITA−/− mice were infected with 10^6^ FFU LCMV WE. At day 15 after infection, the expression of the exhaustion marker PD-1 on LCMV-specific CD8+ T cells in spleen and liver was determined by staining of dextramer+ CD8+ T cells for PD-1. (A) Each dot represents the percentage of PD1+ dextramer+ CD8+ T cells among all dextramer+ CD8+ T cells of spleen or liver of individual mice. (B) Each dot represents the mean fluorescence intensity (MFI) of PD-1 staining of all dextramer+ CD8+ T cells in spleen or liver of individual mice.

To study whether the decreased degranulation capacity of LCMV-specific CD8+ T cells in the livers of CIITA−/− mice was a consequence of T-cell exhaustion, we analyzed the expression of the major exhaustion marker PD-1 on LCMV-specific CD8+ T cells in the liver at day 15 after infection; PD-1 is considered to be the first exhaustion marker expressed by increasingly dysfunctional CD8+ T cells [30]. However, we did not find increased PD-1 expression in the total intrahepatic CD8+ T cell population of CIITA−/− mice (Figure 8A) or in the intrahepatic LCMV-specific dextramer+ CD8+ T cell fraction (Figure 8B), both when determined as percentage of PD-1-expressing cells among the CD8+ T cells (left panels) or as mean fluorescence intensity (MFI) per analyzed cell (right panels). Thus, the CD8+ T-cell dysfunction in acutely LCMV infected CIITA−/− mice is unlikely to be explained by T-cell exhaustion.

## Discussion

Although CD4+ T cells are essential for the generation of memory CD8+ T cells [31] and sustained control of viral infections, their role in the initiation of the adaptive immune response to acute viral infection is controversial [22]. Here, we had used two different methods to induce CD4+ T cell-lymphopenia in mice; one by genetic deficiency of CIITA (Figure S1), the other by treatment with depleting antibody (Figure S2). Both models of CD4+ T cell-lymphopenia manifested protracted viral infection of the liver (Figures 1, 2), indicating that CD4+ T cells are crucial for the initiation of an effective anti-viral immune response during acute viral hepatitis. The paucity of CD4+ T cells seemed to induce insufficient expansion of the intrahepatic CD8+ T-cell pool (Figure 3), most notably of virus-specific CD8+ T cells (Figure 5). Our data does not indicate whether the absence of CD4+ T cells had caused inefficient local expansion of liver-infiltrating CD8+ T cells or inefficient recruitment of CD8+ T cells to infected livers; however, we did not find defective production of essential chemokines for the recruitment into infected liver (Figure 4A) or a general defect of non-specific recruitment of leukocytes (Figure 4B). Nonetheless, it has been reported that the recruitment of CD8+ T cells into infected tissues requires CD4+ T cell help [32]; therefore, this issue requires further investigation. Intriguingly, the reduced expansion of virus-specific CD8+ T cells in the liver not only caused globally impaired anti-viral effector functions, notably production of IFN-γ [33] and degranulation (Figures 6, 7), but also impaired anti-viral functionality of LCMV-specific CD8+ T cells on a per-cell basis (Figure 7B). However, the impaired functionality of virus-specific CD8+ T cells in the livers of CD4+ T cell-lymphopenic mice did not seem to be associated with increased expression of the exhaustion marker PD-1 (Figure 8); PD-1 is the first exhaustion marker to be expressed in a progressing loss of function [31] and expression is linked to viral load [34]. This finding indicated that functional impairment of CD8+ T cells may occur without manifest up-regulation of exhaustion markers, at least during the time-points analyzed in this study.

Our demonstration of a causal relationship between the presence of CD4+ T cells and the outcome of acute viral liver infection is in full agreement with previous findings that had uncovered an association between a broad and strong CD4+ T-cell response to hepatitis viruses and the ability to clear the infection [6]. However, our findings seem to contradict the findings by Thimme et al. [12] who showed that depletion of CD4+ T cells does not significantly influence the clearance of HBV infection in chimpanzees. We think that these discrepant results can be explained by the timing of CD4+ T-cell depletion. Indeed, in the study by Thimme et al. the CD4+ T cells were depleted at day 6 after HBV inoculation, when the initial priming of CD8+ T cells probably had already occurred. In our study, by contrast, the mice were already depleted of CD4+ T cells at the time of virus inoculation and the priming of the CD8+ T-cell response took place in absence of CD4+ T cells.

It is generally believed that CD4+ T cells are not required for the control of acute low-dose LCMV infections (reviewed in [22]), as type I IFN can substitute CD4+ T-cell help in the priming of LCMV-specific CD8+ T cells [14], [35]. Indeed, Matloubian et al. [24] had previously shown that depletion of CD4+ T cells does not significantly influence the clearance of acute infection with LCMV of the strain Armstrong that does, however, not cause hepatitis. Here, in contrast, we infected mice with LCMV-WE, which is more virulent and thus able to induce acute hepatitis [15]. Thus, it is possible that the differential requirement for CD4+ T cells may be related to differential virulence of the LCMV strains. Be that as it may, in the hepatitis-inducing LCMV-WE infection that we have studied here, CD4+ T cell-lymphopenia seemed to induce an impaired intrahepatic CD8+ T-cell response and impaired intrahepatic virus control. Indeed, this functional impairment seemed to be more distinct in the liver than in the spleen (Figure 7), indicating that virus control in the liver depends critically on CD4+ T-cell help.

Taken together, our findings indicate the relevance of CD4+ T cells for virus control in the liver, also during acute viral hepatitis. Our results suggest that ineffective CD4+ T-cell help may promote the evolution of acute liver infection towards chronic viral hepatitis. Therapeutic and vaccine strategies aimed at strengthening acute CD4+ T-cell responses may hence prove useful to prevent the chronification of hepatitis virus infections.

## Supporting Information

Figure S1 **Reduced numbers of CD4+ T cells, but not CD8+ T cells in CIITA−/− mice.** Spleen and liver mononuclear cells of C57BL/6 wild-type and CIITA−/− mice were isolated and stained for CD4+ and CD8+ T cells respectively. Each dot represents the absolute number of CD4+ or CD8+ T cells per spleen or liver of individual mice.
(TIF)

Figure S2 **Efficacy of CD4+ T-cell depletion by anti-CD4 antibody treatment.** C57BL/6 mice were treated twice weekly with depleting anti-CD4 antibody (GK1.5) or isotype-matched control antibody and depletion efficacy was determined by flow cytometry. Shown are the percentages of CD4+ T cells (stained with anti-CD4 antibody of clone RM4-4) among all CD3+ T cells (A) and representative CD4+ staining of individual mice at day 18 of infection (B).
(TIF)

Figure S3 **Liver-infiltrating CD8+ T cell numbers in early LCMV infection.** LCMV-infected C57BL/6 or CIITA−/− mice were assessed for liver-infiltrating CD8+ T cell numbers. Each dot represents the absolute number of CD8+ T cells per liver of one individual mouse at day 7 or 9 after LCMV-infection.
(TIF)

Figure S4 **Analysis of LCMV-specific CD8+ T cell response with LCMV-gp33 loaded H-2D^b^ dextramers.** LCMV-infected C57BL/6 or CIITA−/− mice were assessed for LCMV-specific liver-infiltrating CD8+ T cells that recognize the immunodominant gp33 peptide bound to H-2D^b^ molecules by immunofluorescent staining with gp33 loaded H-2D^b^ dextramers, as assessed by flow cytometry. Shown are representative dextramer stainings of liver-infiltrating CD8+ T cells from mice at day 15 of infection.
(TIF)

Figure S5 **Liver-infiltrating LCMV-specific CD8+ T cell numbers in early LCMV infection.** LCMV-infected C57BL/6 or CIITA−/− mice were assessed for LCMV-specific liver-infiltrating CD8+ T cell numbers by immunofluorescent staining with gp33 loaded H-2D^b^ dextramers. Each dot represents the percentage of dextramer+ CD8+ T cells among CD8+ T cells per liver of one individual mouse at day 7 or 9 after LCMV-infection.
(TIF)

Figure S6 **Analysis of IFN-γ production by CD8+ T cells in response to stimulation with LCMV-gp33 peptide.** Liver-infiltrating CD8+ T cells of LCMV-infected C57BL/6 or CIITA−/− mice were assessed by flow cytometry for IFN-γ production in response to stimulation with the immunodominant LCMV-gp33 peptide. Shown are representative intracellular IFN-γ stainings of liver-infiltrating CD8+ T cells from mice at day 15 of infection.
(TIF)

Figure S7 **Analysis of IFN-γ production by CD8+ T cells in early LCMV-infection.** Liver-infiltrating CD8+ T cells of LCMV-infected C57BL/6 or CIITA−/− mice were assessed by flow cytometry for IFN-γ production in response to stimulation with the immunodominant LCMV-gp33 peptide. Each dot represents the percentage of IFN-γ stained infiltrating CD8+ T cells per liver of one individual mouse at day 7 or 9 of infection.
(TIF)

Figure S8 **Analysis of degranulation capacity of LCMV-gp33 specific CD8+ T cells based on CD107a staining.** Liver-infiltrating LCMV-specific CD8+ T cells of LCMV-infected C57BL/6 or CIITA−/− mice were assessed by flow cytometry for LCMV-gp33 loaded H-2D^b^ dextramers (upper panels). The dextramer+ cells were consecutively gated for CD107a staining as degranulation marker (lower panels). Shown are representative dextramer and CD107a stainings of liver-infiltrating CD8+ T cells from mice at day 15 of infection. The indicated percentage of LCMV-specific CD107a+ cells in the lower panels relates to the dextramer+ cells in the respective parent gates of the upper panels.
(TIF)

Figure S9 **Analysis of degranulation capacity of CD8+ T cells in early infection.** At day 7 or 9 after infection, the degranulation capacity of liver-infiltrating CD8+ T cells (A) or liver-infiltrating LCMV-specific dextramer+ CD8+ T cells (B) in response to stimulation with LCMV-gp33 peptide was determined by staining for CD107a. Each dot represents the percentage of degranulated CD8+ T cells among all CD8+ T cells (A) or among all dextramer+ CD8+ T cells (B) per liver of one individual mouse at day 7 or 9 of infection.
(TIF)
