# Supplementary figures and images for: CD4+ T-Cell Help Is Required for Effective CD8+ T Cell-Mediated Resolution of Acute Viral Hepatitis in Mice
Source: PLoS One. 2014 Jan 21;9(1):e86348. doi: 10.1371/journal.pone.0086348 (PMC3897723; doi:10.1371/journal.pone.0086348)

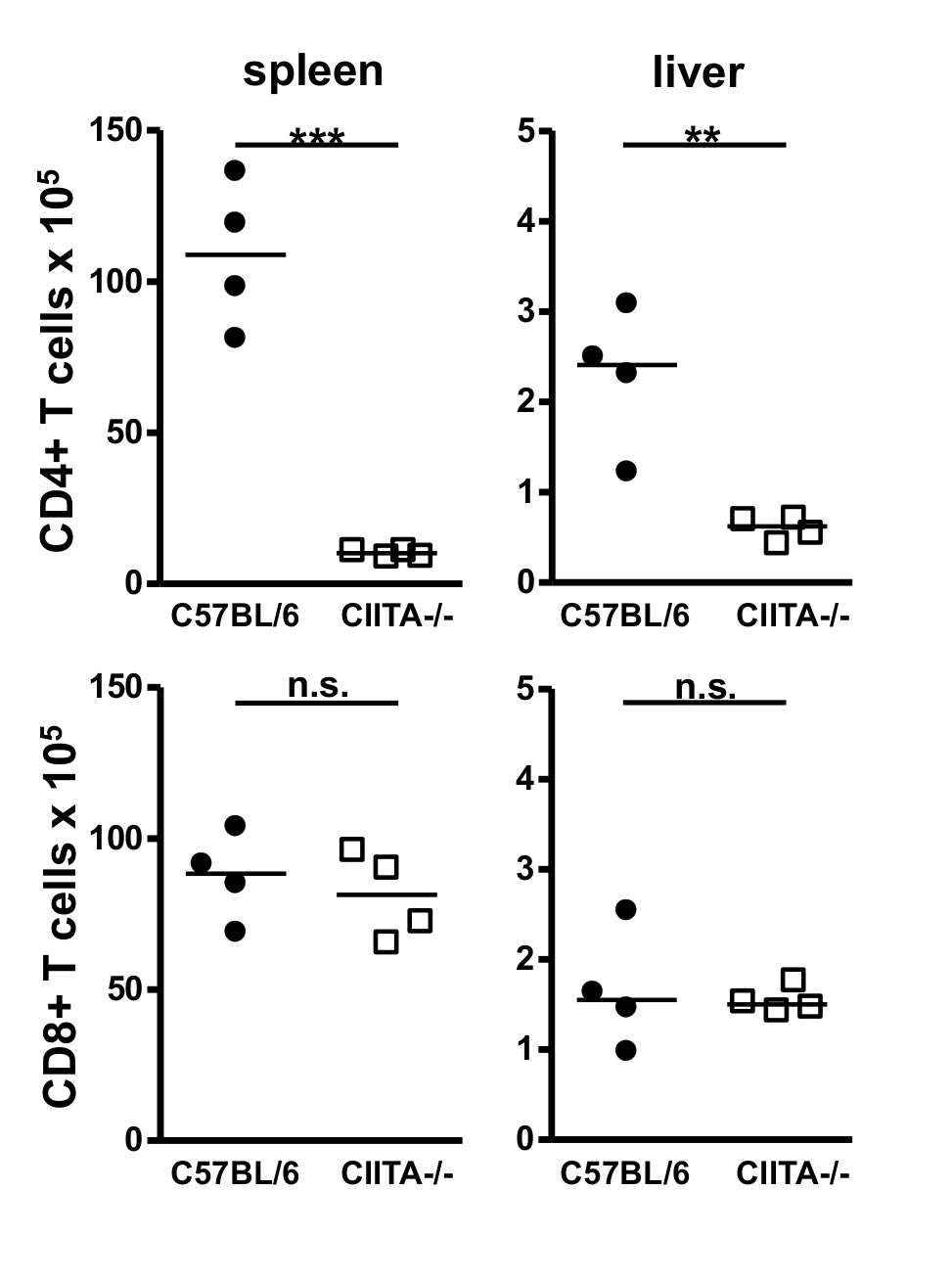

Supplement: Figure S1 — Reduced numbers of CD4+ T cells, but not CD8+ T cells in CIITA−/− mice. Spleen and liver mononuclear cells of C57BL/6 wild-type and CIITA−/− mice were isolated and stained for CD4+ and CD8+ T cells respectively. Each dot represents the absolute number of CD4+ or CD8+ T cells per spleen or liver of individual mice. (TIF) [file pone.0086348.s001.tif]

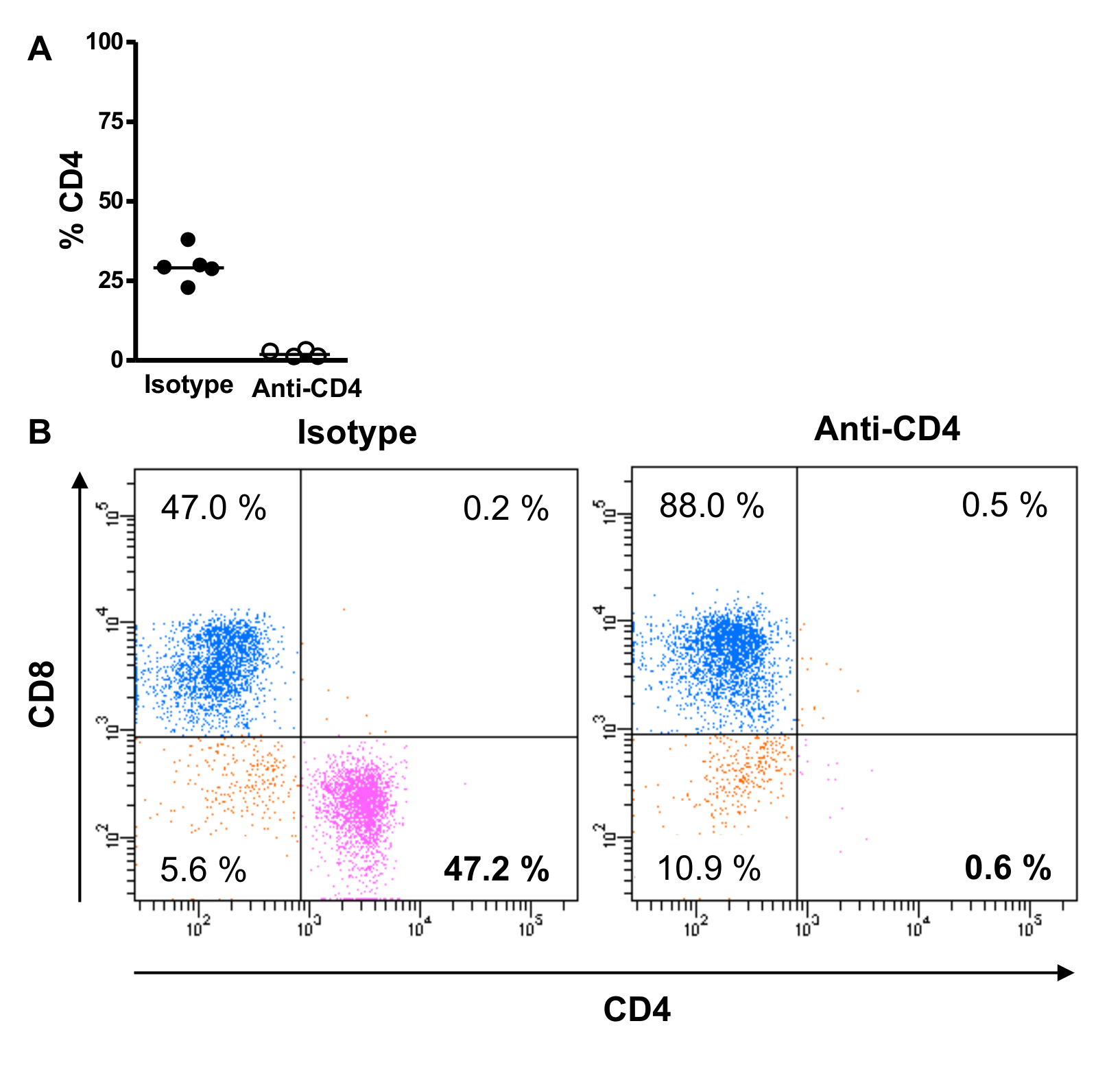

Supplement: Figure S2 — Efficacy of CD4+ T-cell depletion by anti-CD4 antibody treatment. C57BL/6 mice were treated twice weekly with depleting anti-CD4 antibody (GK1.5) or isotype-matched control antibody and depletion efficacy was determined by flow cytometry. Shown are the percentages of CD4+ T cells (stained with anti-CD4 antibody of clone RM4-4) among all CD3+ T cells (A) and representative CD4+ staining of individual mice at day 18 of infection (B). (TIF) [file pone.0086348.s002.tif]

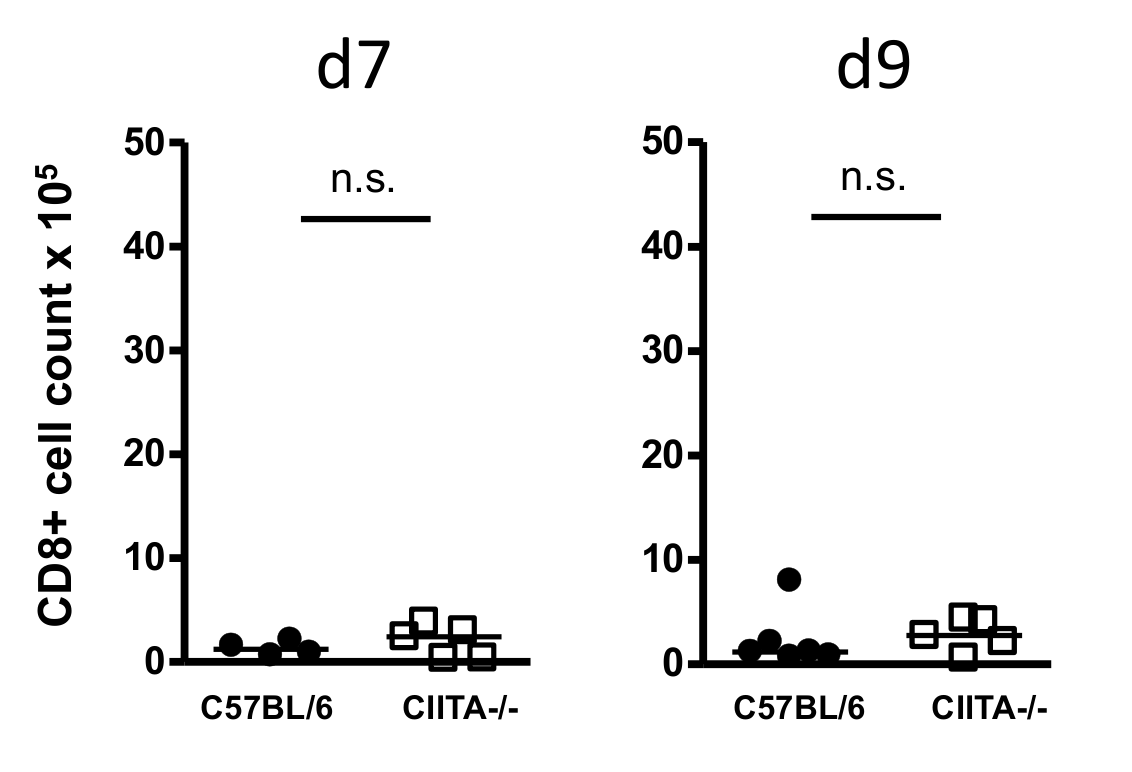

Supplement: Figure S3 — Liver-infiltrating CD8+ T cell numbers in early LCMV infection. LCMV-infected C57BL/6 or CIITA−/− mice were assessed for liver-infiltrating CD8+ T cell numbers. Each dot represents the absolute number of CD8+ T cells per liver of one individual mouse at day 7 or 9 after LCMV-infection. (TIF) [file pone.0086348.s003.tif]

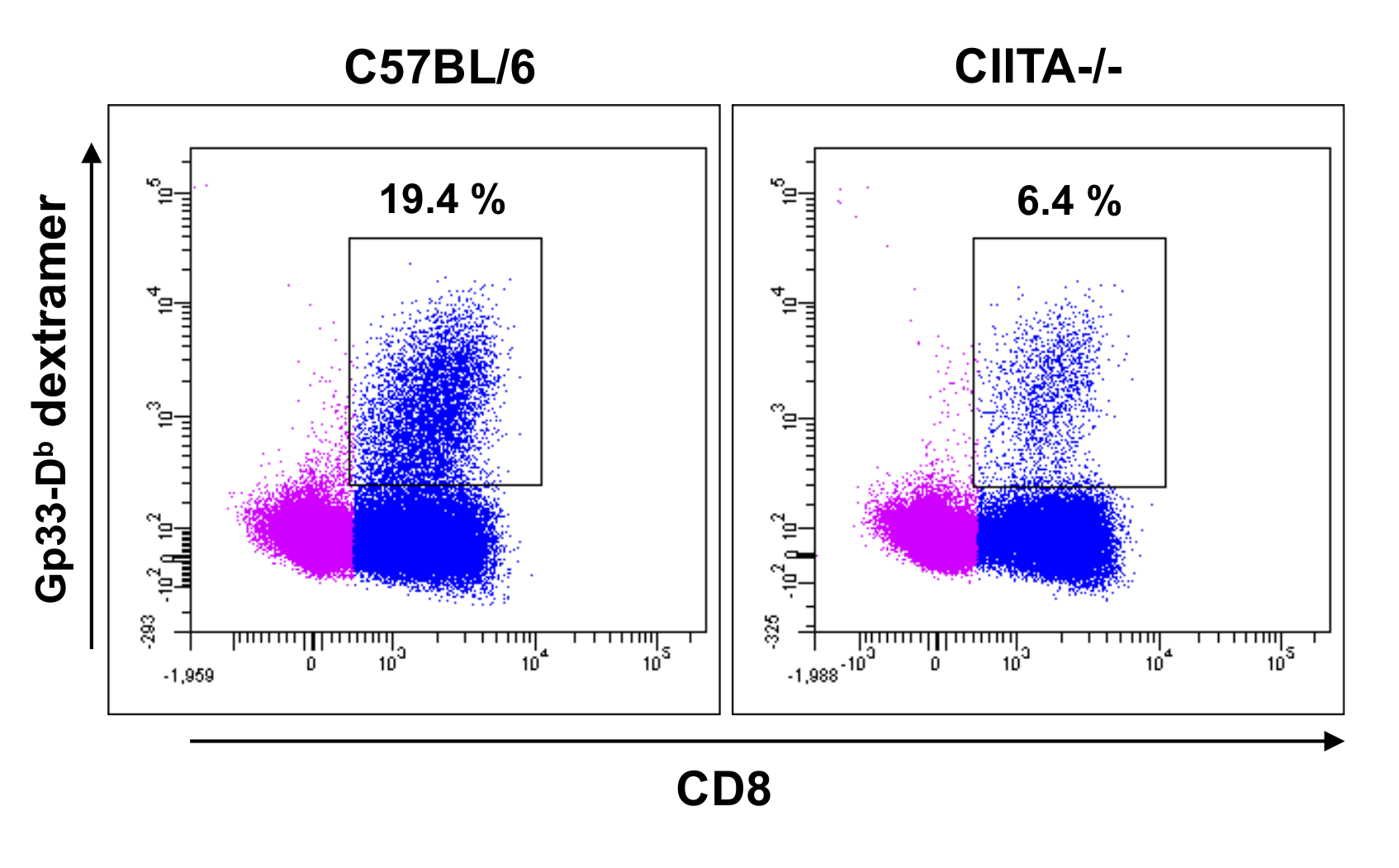

Supplement: Figure S4 — Analysis of LCMV-specific CD8+ T cell response with LCMV-gp33 loaded H-2Db dextramers. LCMV-infected C57BL/6 or CIITA−/− mice were assessed for LCMV-specific liver-infiltrating CD8+ T cells that recognize the immunodominant gp33 peptide bound to H-2Db molecules by immunofluorescent staining with gp33 loaded H-2Db dextramers, as assessed by flow cytometry. Shown are representative dextramer stainings of liver-infiltrating CD8+ T cells from mice at day 15 of infection. (TIF) [file pone.0086348.s004.tif]

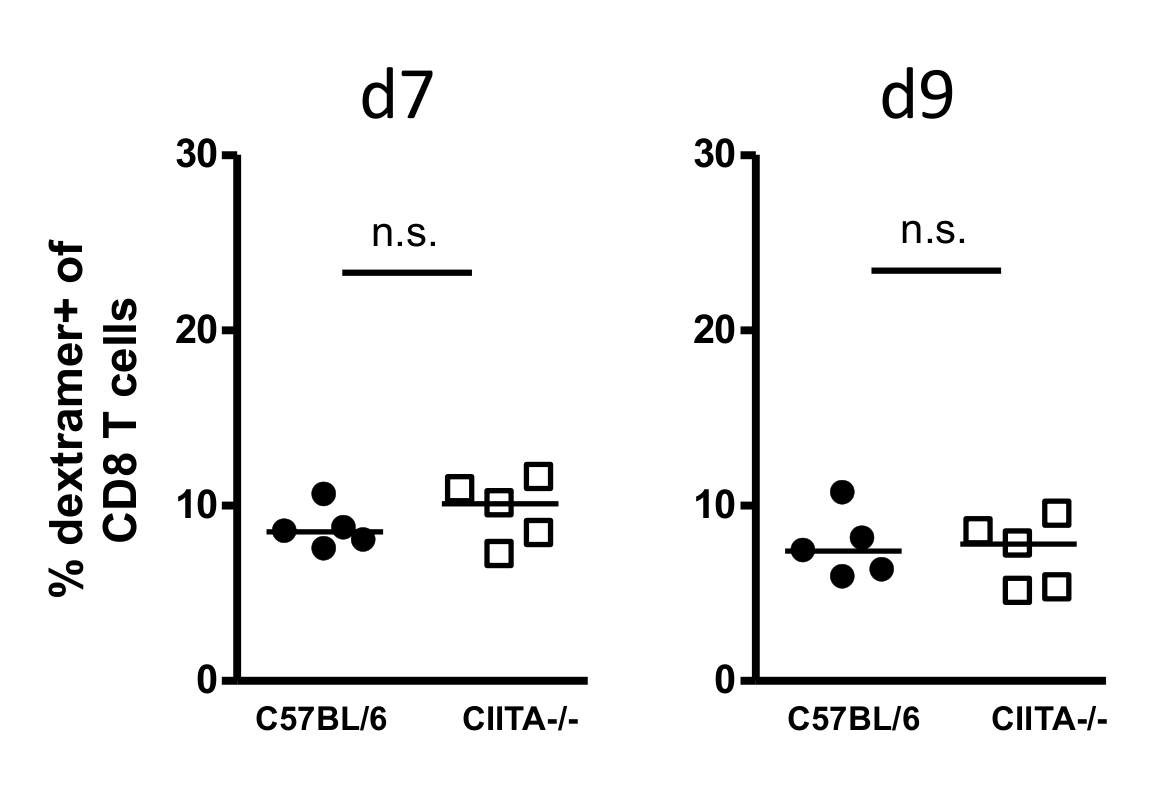

Supplement: Figure S5 — Liver-infiltrating LCMV-specific CD8+ T cell numbers in early LCMV infection. LCMV-infected C57BL/6 or CIITA−/− mice were assessed for LCMV-specific liver-infiltrating CD8+ T cell numbers by immunofluorescent staining with gp33 loaded H-2Db dextramers. Each dot represents the percentage of dextramer+ CD8+ T cells among CD8+ T cells per liver of one individual mouse at day 7 or 9 after LCMV-infection. (TIF) [file pone.0086348.s005.tif]

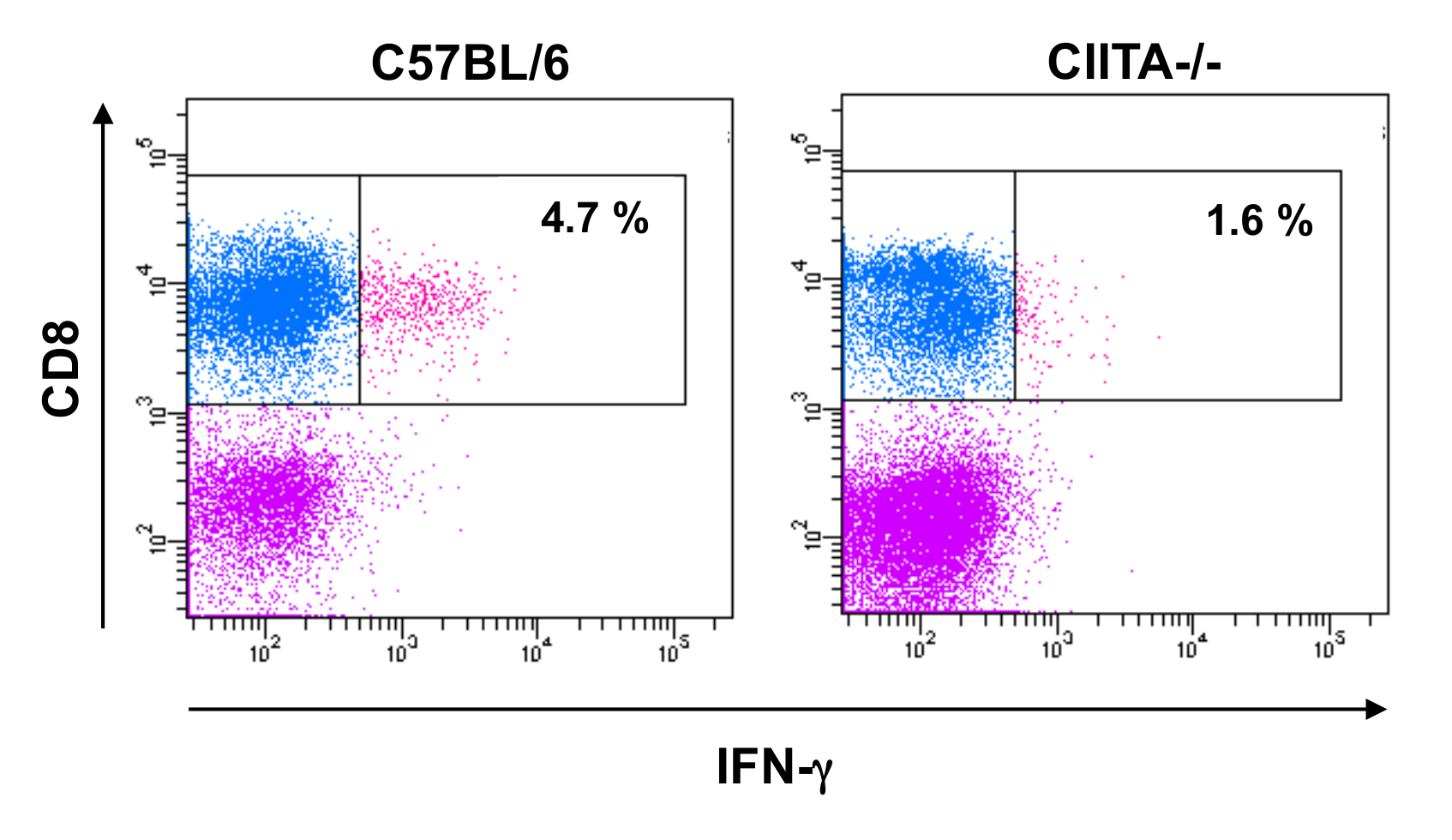

Supplement: Figure S6 — Analysis of IFN-γ production by CD8+ T cells in response to stimulation with LCMV-gp33 peptide. Liver-infiltrating CD8+ T cells of LCMV-infected C57BL/6 or CIITA−/− mice were assessed by flow cytometry for IFN-γ production in response to stimulation with the immunodominant LCMV-gp33 peptide. Shown are representative intracellular IFN-γ stainings of liver-infiltrating CD8+ T cells from mice at day 15 of infection. (TIF) [file pone.0086348.s006.tif]

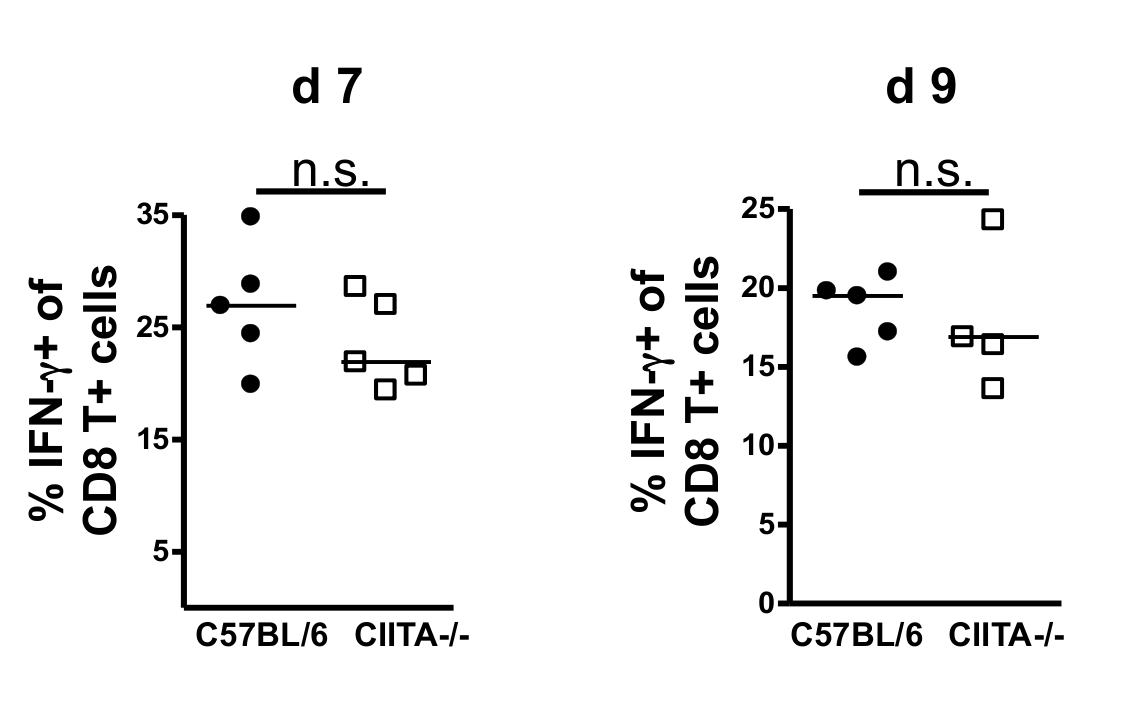

Supplement: Figure S7 — Analysis of IFN-γ production by CD8+ T cells in early LCMV-infection. Liver-infiltrating CD8+ T cells of LCMV-infected C57BL/6 or CIITA−/− mice were assessed by flow cytometry for IFN-γ production in response to stimulation with the immunodominant LCMV-gp33 peptide. Each dot represents the percentage of IFN-γ stained infiltrating CD8+ T cells per liver of one individual mouse at day 7 or 9 of infection. (TIF) [file pone.0086348.s007.tif]

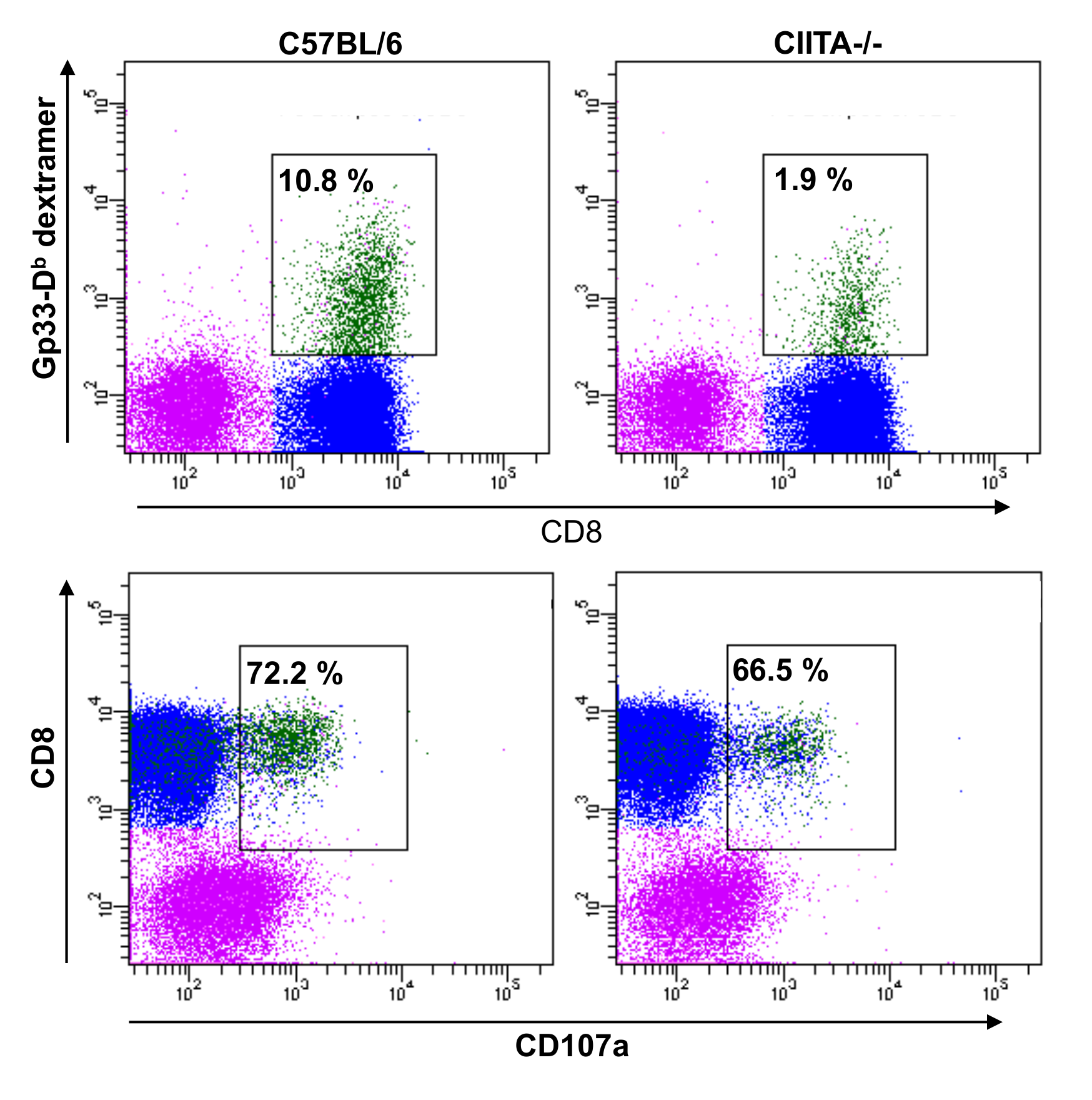

Supplement: Figure S8 — Analysis of degranulation capacity of LCMV-gp33 specific CD8+ T cells based on CD107a staining. Liver-infiltrating LCMV-specific CD8+ T cells of LCMV-infected C57BL/6 or CIITA−/− mice were assessed by flow cytometry for LCMV-gp33 loaded H-2Db dextramers (upper panels). The dextramer+ cells were consecutively gated for CD107a staining as degranulation marker (lower panels). Shown are representative dextramer and CD107a stainings of liver-infiltrating CD8+ T cells from mice at day 15 of infection. The indicated percentage of LCMV-specific CD107a+ cells in the lower panels relates to the dextramer+ cells in the respective parent gates of the upper panels. (TIF) [file pone.0086348.s008.tif]

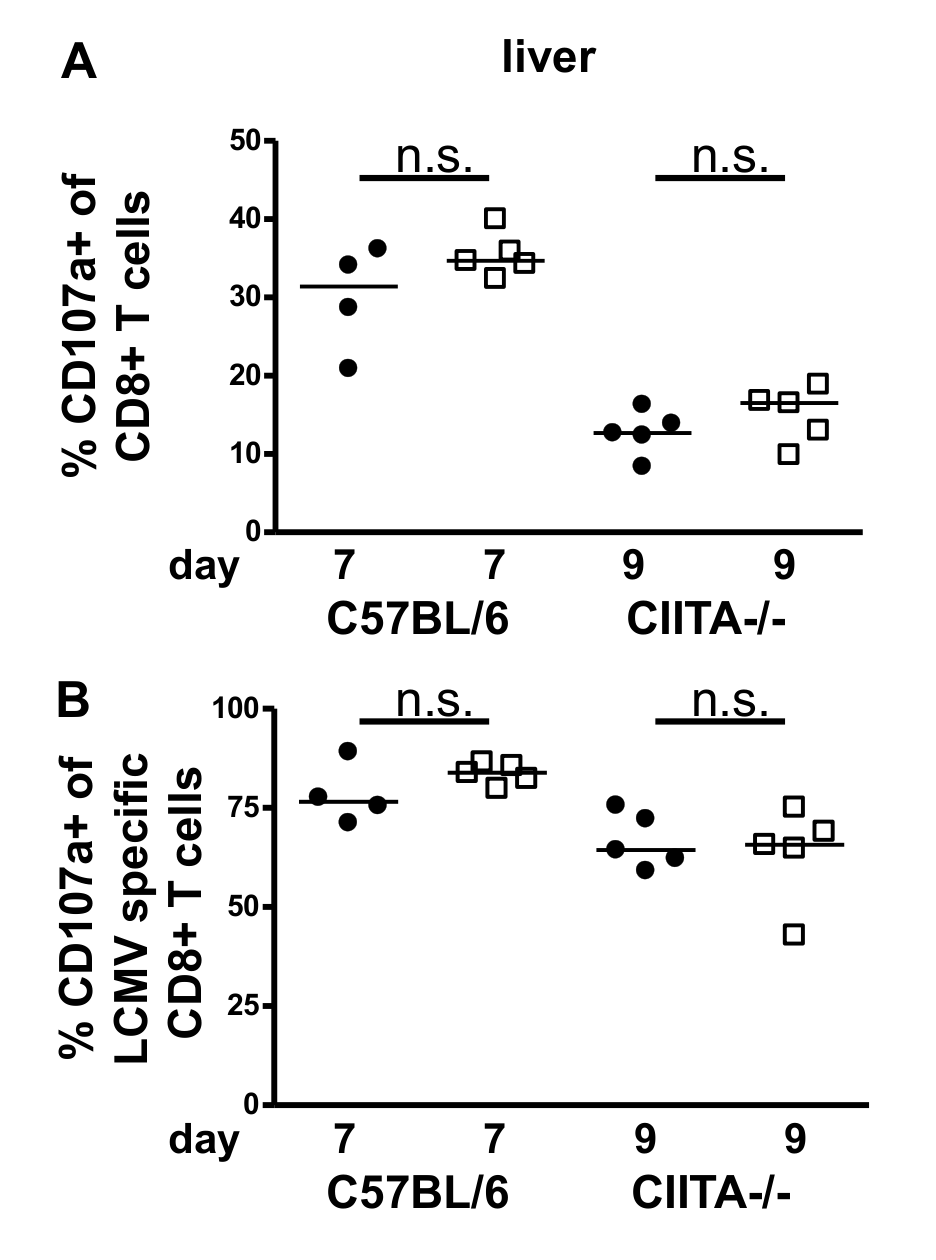

Supplement: Figure S9 — Analysis of degranulation capacity of CD8+ T cells in early infection. At day 7 or 9 after infection, the degranulation capacity of liver-infiltrating CD8+ T cells (A) or liver-infiltrating LCMV-specific dextramer+ CD8+ T cells (B) in response to stimulation with LCMV-gp33 peptide was determined by staining for CD107a. Each dot represents the percentage of degranulated CD8+ T cells among all CD8+ T cells (A) or among all dextramer+ CD8+ T cells (B) per liver of one individual mouse at day 7 or 9 of infection. (TIF) [file pone.0086348.s009.tif]
